# Does wine glass size influence sales for on-site consumption? A multiple treatment reversal design

**DOI:** 10.1186/s12889-016-3068-z

**Published:** 2016-06-07

**Authors:** Rachel Pechey, Dominique-Laurent Couturier, Gareth J. Hollands, Eleni Mantzari, Marcus R. Munafò, Theresa M. Marteau

**Affiliations:** Behaviour and Health Research Unit, University of Cambridge, Cambridge, UK; MRC Integrative Epidemiology Unit (IEU), UK Centre for Tobacco and Alcohol Studies, School of Experimental Psychology, University of Bristol, Bristol, UK

**Keywords:** Alcohol, Glass size, Sales, Choice architecture

## Abstract

**Background:**

Wine glass size can influence both perceptions of portion size and the amount poured, but its impact upon purchasing and consumption is unknown. This study aimed to examine the impact of wine glass size on wine sales for on-site consumption, keeping portion size constant.

**Methods:**

In one establishment (with separate bar and restaurant areas) in Cambridge, England, wine glass size (Standard; Larger; Smaller) was changed over eight fortnightly periods. The bar and restaurant differ in wine sales by the glass vs. by the bottle (93 % vs. 63 % by the glass respectively).

**Results:**

Daily wine volume purchased was 9.4 % (95 % CI: 1.9, 17.5) higher when sold in larger compared to standard-sized glasses. This effect seemed principally driven by sales in the bar area (bar: 14.4 % [3.3, 26.7]; restaurant: 8.2 % [−2.5, 20.1]). Findings were inconclusive as to whether sales were different with smaller vs. standard-sized glasses.

**Conclusions:**

The size of glasses in which wine is sold, keeping the portion size constant, can affect consumption, with larger glasses increasing consumption. The hypothesised mechanisms for these differential effects need to be tested in a replication study. If replicated, policy implications could include considering glass size amongst alcohol licensing requirements.

**Trial registration:**

ISRCTN registry: ISRCTN12018175. Registered 12^th^ May 2015.

## Background

Alcohol consumption is ranked 5th amongst the 20 leading risk factors for global burden of disease [1]. In addition to price, availability and marketing [2–4], other cues may also encourage people to drink more than they might otherwise, such as glassware and portion size. A recent Cochrane systematic review provided evidence that larger portion, package and tableware size increase consumption of food and non-alcoholic beverages [5]. Whilst this review did not identify any studies concerning the impact of these cues on alcohol consumption, it seems reasonable to expect that serving wine in larger portions, bottles and glassware will increase its consumption. The mechanisms underlying the “portion size effect” are not fully understood. People generally perceive the amount served to them as representing an appropriate portion size and consume less when offered smaller portions and more when offered larger portions [6].The portion sizes, therefore, we routinely encounter can shape the social and personal norms for what we consider a suitable amount to consume [7]. The amount of food or the size of a non-alcoholic drink in front of us can also influence the size of bites or sips taken, with larger quantities resulting in larger bites or sips [8, 9]. The way in which food and drink is presented can also influence consumption. For example, the size and shape of a plate or glass can alter perceptions of quantity and influence how much is served [10, 11]. These effects may often operate outside of awareness [12, 13] making smaller default sizes for portions, packages and tableware effective barriers to the overconsumption that larger sizes cue [14].

The aim of the current study is to generate preliminary evidence of the impact of wine glass size on sales of wine for on-site consumption in one UK bar/restaurant. Wine in the UK can be purchased either by the glass, with a set portion (125 ml (must be available), 175 ml or 250 ml), or by the bottle (standard size: 750 ml). The mechanisms underlying any effect of glass size on consumption may differ according to whether it is bought and consumed by the bottle or by the glass [12, 15, 16]. Glasses provided alongside bottles may affect the actual portion served (by influencing the amount poured) [17–19], and glasses containing pre-served portions may change perceptions of portion size [20, 21] and in turn how much is consumed. However, such effects may be curbed if people drink a certain number of glasses of wine regardless of the perceived size of the glasses [22–24]. This study reports the effects of wine glass size on wine sales, examining the results within separate bar and restaurant areas of one UK establishment –– where sales varied in terms of the extent to which these occurred by the glass or by the bottle.

## Methods

### Setting

The study was conducted in an independent eating and drinking establishment in Cambridge, England, from mid-March to early July 2015. The establishment had separate bar and restaurant areas, both selling food and drink, where wine could be purchased by the glass (125 ml or 175 ml), bottle (750 ml) or carafe (500 ml or 1000 ml). All wine purchases were usually served in the establishment’s standard glass, which had a 300 ml capacity. During the study period, sales by the glass accounted for around 78 % of total wine transactions in the establishment. Approximately 90 % of wine sold by the glass was in 175 ml portions, with the remaining 10 % being 125 ml portions. The establishment sold on average 126.6 litres of wine per week (S.D. 14.9 litres) for the equivalent period in 2014 (9.9 % of their total sales); the equivalent figure for the study period was 121.0 litres (S.D. 12.6). The average price of a 175 ml glass of wine in the establishment for the study period was around £5 (€7/$7.50) (UK average £3.46 (€5/$5) [25]).

### Design

The study design involved repeated baseline and intervention phases, with each of eight consecutive periods lasting two weeks:

A: Baseline: standard 300 ml glass used

B: Larger 370 ml glass replaced standard glass

A: Standard 300 ml glass used

C: Smaller 250 ml glass replaced standard glass

B: Larger glass

A: Standard glass

C: Smaller glass

A: Standard glass

The primary outcome was the daily volume of wine (ml) purchased in ‘Larger glass’ and ‘Smaller glass’ intervention periods compared to baseline (all ‘A’ periods).

### Procedure

Three different sized but similarly shaped wine glasses were used in the establishment for each of eight fortnightly periods. The glasses were used both when serving wine by the glass and when providing glasses for purchases of bottles or carafes of wine (i.e. without changing portion sizes). In keeping with their license to sell alcohol, wine served by the glass was measured out by bar staff using CE stainless steel thimbles (Beaumont ^tm^) which, when filled to the brim, contained the volume specified on the thimble (i.e. 125 ml or 175 ml). Glasses not being used in that fortnightly period were removed from the establishment’s shelves, so that only the appropriate glass size was available. There were no changes to the establishment’s wine menu or pricing during the study period.

**Fig. 1:**
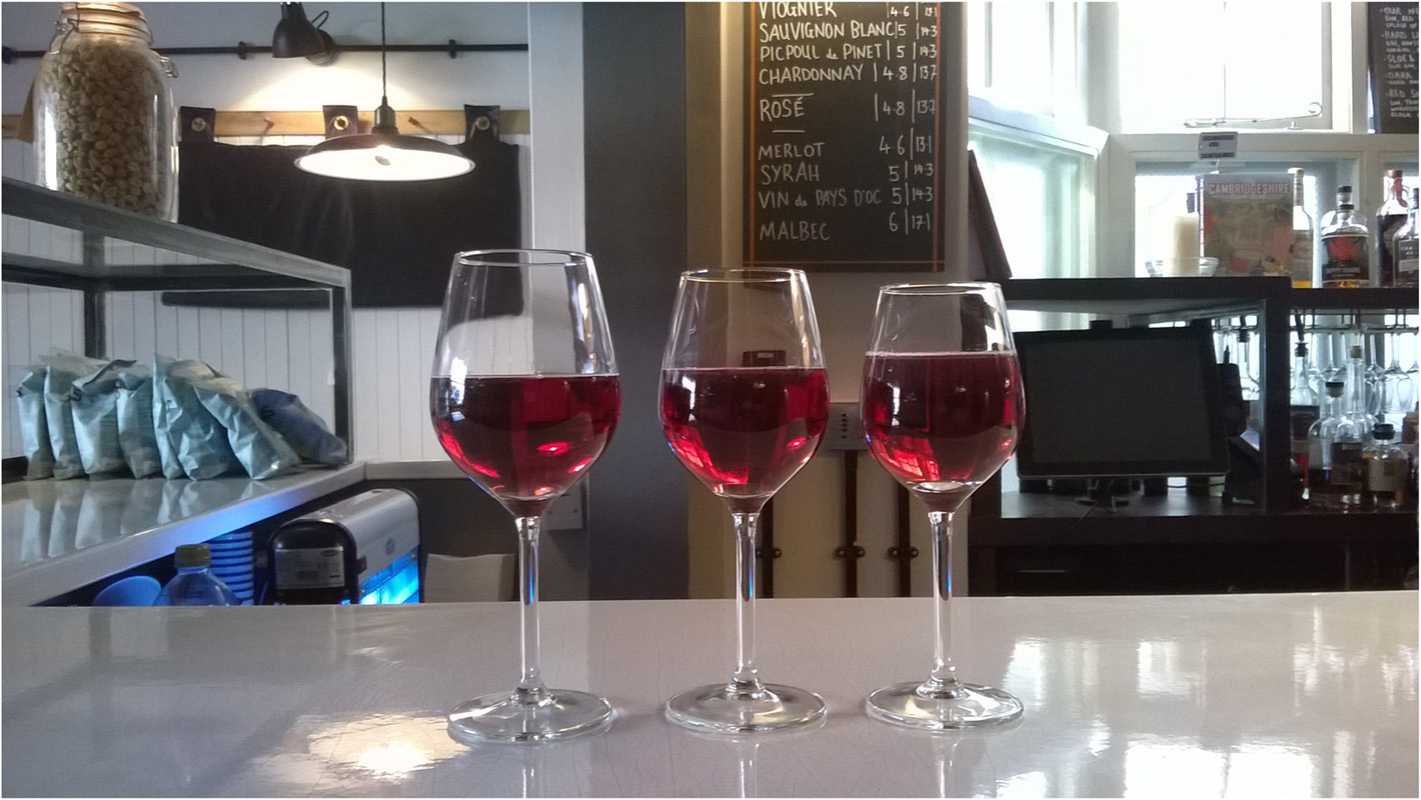
Design of glasses used in the study (filled to 175 ml)

The standard glasses used in the target establishment were unlined Royal Leerdam Fortius glasses (see Fig. 1), with a capacity of 300 ml. The two additional glasses were: Smaller: 250 ml glass of same design; Larger: 370 ml glass of same design.

Sales data were obtained from the establishment’s till records. Daily wine volume (ml) sold was calculated by multiplying each volume available to purchase (e.g., 175 ml glasses, 750 ml bottles) by the number of units sold daily, and summing across all wine purchases.

### Analysis

Regression analyses predicted daily volume of wine (ml) purchased from glass size. Analyses included dummy variables as covariates for day of the week, overall sales in the establishment each day (excluding wine sales), and maximum daily temperature in Cambridge [26]. Equivalent wine sales in the previous year (allowing adjustment for seasonal fluctuations in sales), holiday periods (two dummies indicating school holidays and public holidays) were also explored as covariates, but were non-significant and led to worse model predictions, so were excluded from final analyses. The number of items sold (excluding wine sales) for each day was used as a proxy for the number of customers, to control for sales fluctuations over time. Both wine sales and sales excluding wine were logged in the analyses. Due to heteroscedasticity, both the mean and variance of sales (see Table 2) were included in models (using identity and log links respectively).

Analyses were also conducted separately for the bar and restaurant areas of the establishment, given the differential distribution of wine sales by the glass in the two areas (bar: 93 % by the glass; restaurant: 63 % by the glass).

## Results

Table 1 presents the unadjusted mean daily volumes of wine sold, overall and in the bar and restaurant areas. The raw data here suggest higher sales on days when using larger glasses compared to days using standard glasses, with an inconsistent pattern for smaller glasses. However, this does not control for potential confounders, such as establishment busyness, with, for example, the first fortnight when larger glasses were used overlapping with the Easter weekend.

**Table 1:** Mean (s.d) daily volume sold in ml by glass size and location

	Small	Standard	Large
Overall	16,681	16,624	18,993
	(8,437)	(8,122)	(8,090)
Bar	6,791	5,989	7,235
	(4,063)	(3,634)	(3,809)
Restaurant	9,890	10,635	11,757
	(4,819)	(5,117)	(4,837)

**Fig. 2:**
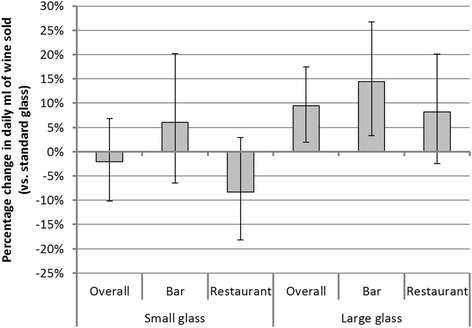
Percentage change in daily ml of wine sold, compared to sales with the standard glass (error bars show 95 % CIs). All regressions controlled for day of the week, overall sales (excluding wine sales) in the establishment each day and maximum daily temperature in Cambridge. Standard glasses were the reference group for glass size. Coefficients and CIs were back-transformed (using 100*[exp(B)-1]), to reflect percentage change in daily ml of wine sold when using different glass sizes compared to the standard glass

The results of the regression analyses are presented in Table 2. Figure 2 shows the effect of the glass size on sales expressed in percentage. It suggests that there was an effect of larger glass size (compared to standard glass size) overall and in the bar area, with increases in daily wine sales of 9.4 % (95 % CI: 1.9, 17.5) and 14.4 % (95 % CI: 3.3, 26.7) respectively. In the restaurant area, a similar direction of effect was seen but was not significant (8.2 %; 95 % CI: −2.5, 20.1). Findings were inconclusive as to whether wine sales were different with smaller vs. standard glasses.

**Table 2:** Regression models assessing the impact of wine glass size on volume of wine sold^a^

	Bar & Restaurant Estimate (95 % CI) [*p*-value]	Bar Estimate (95 % CI) [*p*-value]	Restaurant Estimate (95 % CI) [*p*-value]
Modelling of the mean (identity link):			
(Intercept)	4.027 (2.644; 5.411) [<0.001]***	3.869 (2.295; 5.443) [<0.001]***	5.027 (3.402; 6.652) [<0.001]***
Busyness level (log)	0.815 (0.623; 1.008) [<0.001]***	0.774 (0.539; 1.009) [<0.001]***	0.677 (0.422; 0.931) [<0.001]***
Monday	−0.044 (−0.143; 0.056) [0.389]	−0.025 (−0.188; 0.139) [0.767]	−0.149 (−0.298; 0.000) [0.052].
Tuesday	−0.113 (−0.199;-0.028) [0.011]*	−0.202 (−0.337;-0.066) [0.005]**	−0.102 (−0.219; 0.015) [0.092].
Wednesday	0.014 (−0.082; 0.110) [0.777]	0.040 (−0.070; 0.150) [0.481]	−0.063 (−0.218; 0.091) [0.423]
Thursday	−0.002 (−0.114; 0.110) [0.973]	0.003 (−0.147; 0.154) [0.965]	−0.059 (−0.238; 0.120) [0.522]
Friday	0.134 (0.024; 0.244) [0.019]*	0.071 (−0.128; 0.271) [0.486]	0.283 (0.167; 0.398) [<0.001]***
Saturday	0.177 (0.049; 0.304) [0.008]**	0.231 (0.049; 0.414) [0.015]*	0.309 (0.171; 0.448) [<0.001]***
Sunday	−0.166 (−0.246;-.085) [<0.001]***	−0.119 (−0.234;-0.004) [0.045]*	−0.219 (−0.345;-0.093) [0.001]***
Small glass	−0.021 (−0.108; 0.066) [0.632]	0.058 (−0.068; 0.184) [0.367]	−0.087 (−0.201; 0.028) [0.143]
Large glass	0.090 (0.019; 0.161) [0.015]*	0.134 (0.033; 0.236) [0.011]*	0.079 (−0.025; 0.183) [0.140]
Temperature	−0.009 (−0.017;-0.001) [0.023]*	−0.017 (−0.028;-0.007) [0.002]**	−0.005 (−0.015; 0.005) [0.310]
Modelling of the variance (log link):			
(Intercept)	−1.560 (−1.753;-.367) [<0.001]***	−1.123 (−1.328;-.919) [<0.001]***	−1.222 (−1.415;-.029) [<0.001]***
Small glass	−0.053 (−0.408; 0.301) [0.769]	−0.111 (−0.466; 0.243) [0.540]	−0.179 (−0.527; 0.168) [0.315]
Large glass	−0.638 (−0.996;-0.279) [0.001]***	−0.745 (−1.156;-0.333) [0.001]***	−0.451 (−0.809;-0.093) [0.015]*
Monday	−0.358 (−0.705;-0.010) [0.047]*	−0.038 (−0.375; 0.298) [0.823]	0.051 (−0.287; 0.390) [0.767]
Tuesday	−0.071 (−0.395; 0.253) [0.670]	0.160 (−0.178; 0.497) [0.356]	−0.193 (−0.525; 0.139) [0.258]
Wednesday	0.145 (−0.181; 0.471) [0.386]	−0.257 (−0.614; 0.099) [0.160]	0.325 (−0.003; 0.652) [0.055].
Thursday	0.431 (0.102; 0.760) [0.012]*	0.325 (−0.008; 0.658) [0.059].	0.510 (0.186; 0.834) [0.003]**
Friday	0.099 (−0.246; 0.443) [0.576]	0.464 (0.116; 0.811) [0.010]*	−0.172 (−0.516; 0.172) [0.329]
Saturday	−0.319 (−0.656; 0.018) [0.067].	−0.613 (−0.956;-0.270) [0.001]***	−0.491 (−0.818;-0.164) [0.004]**
Sunday	0.073 (−0.302; 0.447) [0.705]	−0.039 (−0.415; 0.336) [0.838]	−0.029 (−0.392; 0.334) [0.875]

Significance key: ‘.’ for *p*-value < 0.1, ‘*’ for *p*-value < 0.05, ‘**’ for *p*-value < 0.01, ‘***’ for *p*-value < 0.001

^a^The outcome is the daily volume of wine sold on log scale. ‘Treatment’ contrasts with standard glass as reference were used for the glass size predictor. ‘Sum’ contrasts were used for the days of week. Parameter 95 % confidence intervals and *p*-values respectively appear in parenthesis and in square bracket

## Discussion

Consistent with evidence from the recent Cochrane systematic review that tableware size influences consumption of food and non-alcoholic beverages [5], the larger glass size had an effect on overall wine sales (compared to standard glasses). However, results were inconclusive for the smaller glass size. Separating out the results in the different areas of the establishment, the effect for larger glasses was significant in the bar area, but not in the restaurant area.

Almost all sales in the bar were by the glass. As glasses containing pre-served portions may change perceptions of portion size [20, 21], the results for the larger glasses in the bar area are consistent with larger glasses being perceived to contain less, given the same actual portion [21]. As a result, portions may be drunk faster [20], which may lead people to drink more. Alternatively, there may be decreased satisfaction with perceived-to-be smaller portions or a perception that the portion did not comprise a full glass, leading to additional purchases. However, these mechanisms might be expected to operate across glass sizes (i.e., we should observe similar patterns when comparing the smaller and standard glasses), which was not evident in this study. This requires further investigation as to which glass comparisons yield similar results. Additional field studies are warranted alongside laboratory studies to test the two hypotheses for the observed effect outlined above. These findings can be considered within the broader literature of the many sensory and behavioural cues that influence the consumption of alcohol, of which size is just one [27, 28]. Future research might ultimately attempt to elucidate the combination of cues that reduced alcohol consumption the most.

The effect size of the larger wine glasses in the restaurant area, where sales by bottles and carafes represented 1/3 of the sales but 2/3 of the volume, while they represented 1/15 of the sales and 1/5 of the volume in the bar, did not reach statistical significance. This may reflect a smaller effect size of wine glass size in this context that the study was insufficiently powered to detect. Replication of the current study is needed in other settings where wine is served primarily using bottles or carafes in studies powered to detect smaller but potentially important effect sizes.

### Strengths and limitations

This study is the first, to our knowledge, to explore the impact of glass size on wine sales for on-site consumption in a real-world setting (although it is likely that industry research exists). Examining how wine sales were affected in the bar area, where sales were predominantly by the glass, suggests several possible mechanisms that might underlie the influence of glass size, as described above.

However, several limitations should be noted. First, the multiple treatment reversal design has a higher risk of bias than an experimental design. Second, the predominance of sales by the glass in both the bar and restaurant areas meant that we were unable to robustly examine the effects of sales by the bottle. Third, the study took place in only one establishment in a relatively affluent English city. Finally, our outcome measure was sales for on-site consumption rather than consumption itself, although this still represents an objective measure of behaviour.

### Implications for research and policy

Replication of the current intervention is needed. Further field studies could reduce further the risk of bias through experimental designs involving more observations, conducted in settings that include less affluent areas. Further investigations need to establish the contexts in which the strongest effects are likely to occur, including the extent to which these results might extend to in-home alcohol consumption. While further research is needed to establish the reliability of these findings – and in particular, explore the use of different glass sizes – the results offer initial evidence that reducing the use of larger glasses may reduce consumption of alcohol. If further work does suggest glass size might be an effective target for intervention, the next step would be to explore how this might be implemented [29]; for example, ensuring that all glasses were below a certain size could be one criterion amongst alcohol licensing requirements.

## Conclusions

In summary, the size of glasses in which wine is sold, keeping the portion size constant, affected wine sales, but only when comparing larger glasses to standard glasses. The possible mechanisms for these effects need to be assessed in addition to replication of the current study in other establishments.

### Ethics, consent and permissions

Ethical approval for the study was obtained from the University of Cambridge’s Psychology Research Ethics Committee (Ref: Pre.2014.127).

### Consent for publication

Consent was obtained from the establishment for their participation and for publication of the results.

### Availability of data and materials

The data are commercially sensitive, provided by the establishment on condition that they were not shared beyond the authors.
